# How Old Is Old? An Age-Stratified Analysis of Elderly Liver Donors above 65

**DOI:** 10.3390/jcm11133899

**Published:** 2022-07-04

**Authors:** Philipp Houben, Eike Bormann, Felicia Kneifel, Shadi Katou, Mehmet Haluk Morgül, Thomas Vogel, Ralf Bahde, Sonia Radünz, Andreas Pascher, Hartmut Schmidt, Jens Gunther Brockmann, Felix Becker

**Affiliations:** 1 Department of General, Visceral and Transplant Surgery, University Hospital Münster, 48149 Münster, Germany; felicia.kneifel@ukmuenster.de (F.K.); shadi.katou@ukmuenster.de (S.K.); haluk.morguel@ukmuenster.de (M.H.M.); thomas.vogel@ukmuenster.de (T.V.); bahder@web.de (R.B.); sonia.raduenz@uk-essen.de (S.R.); andreas.pascher@ukmuenster.de (A.P.); brockmannjgb@me.com (J.G.B.); felix.becker@ukmuenster.de (F.B.); 2 Institute of Biostatistics and Clinical Research, University Hospital Münster, 48149 Münster, Germany; eike.bormann@ukmuenster.de; 3 Department of Internal Medicine B, Gastroenterology and Hepatology, University Hospital Münster, 48149 Münster, Germany; hartmut.schmidt@uk-essen.de

**Keywords:** age, old donor, liver transplantation, elderly donor, risk stratification, ischemia-reperfusion injury

## Abstract

In liver transplantation, older donor age is a well-known risk factor for dismal outcomes, especially due to the high susceptibility of older grafts to ischemia-reperfusion injury. However, whether the factors correlating with impaired graft and patient survival following the transplantation of older grafts follow a linear trend among elderly donors remains elusive. In this study, liver transplantations between January 2006 and May 2018 were analyzed retrospectively. Ninety-two recipients of grafts from donors ≥65 years were identified and divided into two groups: (1) ≥65–69 and (2) ≥ 70 years. One-year patient survival was comparable between recipients of grafts from donors ≥65–69 and ≥70 years (78.9% and 70.0%). One-year graft survival was 73.1% (donor ≥65–69) and 62.5% (donor ≥ 70), while multivariate analysis revealed superior one-year graft survival to be associated with a donor age of ≥65–69. No statistically significant differences were found for rates of primary non-function. The influence of donor age on graft and patient survival appears not to have a distinct impact on dismal outcomes in the range of 65–70 years. The impact of old donor age needs to be balanced with other risk factors, as these donors provide grafts that offer a lifesaving graft function.

## 1. Introduction

In orthotopic liver transplantation (oLT), donor organ shortage to a variable extent is a challenge for many centers and procurement organizations. The unmet need for life-sustaining grafts demands the utilization of so-called extended criteria donors, e.g., those with pre-existing diseases, donation after cardiac arrest or cardiac death, prolonged intensive care stay, etc. [1]. Among those, elevated donor age is a matter of ongoing debate. Usage of older grafts is complicated by their reduced capacity for recovery and their high susceptibility to ischemia-reperfusion injury. Donor age has been shown to negatively impact the outcome of oLT linear over the entire age range in large enough studies and is part of the widely accepted donor risk index (DRI) [2,3,4]. Internationally, the maximum accepted donor age in deceased donors differs substantially. Lately, the utilization of older donors has captured the focus of discussion in the United States. Halazun et al. demonstrated a very unbalanced utilization of livers of donors exceeding 70 years in the various United Network for Organ Sharing (UNOS) regions [5]. At the same time, the utilization of septuagenarian and octogenarian donors has become common practice in other regions worldwide [3]. This discrepancy reflects the ongoing debate on the risks and prospects of elderly donors in oLT. Advanced donor age has been shown to add overproportioned risk in hepatitis c virus (HCV)-positive recipients before the advent of direct antiviral acting agents (DAA) [6]. Hepatocellular carcinoma (HCC) recipients seem to be less affected by the observed reduced graft survival of older donors in general [2]. Haugen et al. recently demonstrated that refusal of a graft on account of the donor’s old age leads to inferior survival prospects for the potential recipient [7]. The aforementioned insights and findings are helpful in the allocation process if an elderly graft is offered. Nevertheless, center-specific and individual circumstances may contribute to the effects on the outcome of oLT recipients of elderly grafts. This retrospective single-center analysis was consequently designed to identify the risks and potential benefits by utilizing grafts from donors exceeding 64 years of age.

## 2. Materials and Methods

### 2.1. Study Design and Study Population

All adult patients who underwent oLT at the Department of General, Visceral and Transplant Surgery, University Hospital Münster, Germany between January 2006 and May 2018 were screened for inclusion. Ninety-two patients with donors ≥ 65 years were identified and included in the final analysis. Based on the donor age, the eligible patients were further stratified into two groups: (1) ≥65–69 and (2) ≥70 years. The study design was a retrospective single-center study with a follow-up period of 12 months. The study was conducted as per the ethical principles stated in the Declaration of Helsinki and all patients provided consent for the routine recording of clinical data. Specific study approval was obtained from the local ethics committee (Ethik-Kommission der Ärztekammer Westfalen-Lippe und Westfälischen Wilhelms-Universität, No. *2019-473-f-S*). All retrospectively collected data were from patients’ charts, Eurotransplant Network Information System (ENIS), or in-house transplant data files. Before analysis, all data were de-identified. All allografts were procured from donation after brain death donors.

### 2.2. Demographic Data

Baseline donor parameters were obtained from ENIS and included age, gender, body mass index (BMI) as well as donor center (national/international), and a donor risk index (DRI) was calculated, as described [4]. Baseline datasets for eligible recipients included age, gender, BMI, a model for end-stage liver disease (MELD) score, indication for oLT, high urgency (HU) status, hepatitis C status, cold and warm ischemia time, as well as the number of prior transplants. Indication for oLT was defined as the underlying cause/disease and classified into the following indications: acute liver failure (ALF), hepatocellular carcinoma (HCC), viral hepatitis, cholangitis (including primary sclerosing cholangitis (PSC), primary biliary cholangitis (PBC) and secondary sclerosing cholangitis (SSC)), alcoholic cirrhosis, polycystic liver disease (PLD), and others (including Caroli disease, hemochromatosis, amyloidosis, and cryptogenic cirrhosis).

### 2.3. Outcome Measures

The primary outcome for this study was one-year patient survival, estimated by the Kaplan–Meier methodology and compared using Log-Rank testing. The secondary outcome parameters encompassed one-year overall and death-censored graft survival, as well as 30-day and 90-day patient and graft survival, primary non-function (PNF, graft failure (excluding any identifiable cause such as rejection and/or vascular thrombosis) resulting in death or re-transplantation within 30 days of the initial oLT). Additional secondary outcome measures were the rates of biopsy-proven acute rejections (BPAR), early allograft dysfunction as defined by Olthoff et al. [8], peak serum values of alanine transaminase (ALT) and aspartate transaminase (AST), length of stay in an intensive care unit (ICU), length of stay in the hospital, death during initial hospitalization, number, and length of readmissions (not oLT specific) following discharge, frequency of ischemic-type biliary lesions (ITBL) and rates of re-transplantations. All re-transplantations within the observational period of 12 months were counted as a complication for the respective group of the initial oLT and not as an addition oLT case for the respective group, as reported previously [9,10].

### 2.4. Statistical Analysis

Continuous variables were presented as mean and standard deviation and the number of reoperations and the length of readmissions as median (minimum and maximum). Categorial variables were presented as absolute and relative frequencies. Comparisons between the two groups were done either by Wilcoxon rank-sum test for continuous variables or using the Fisher exact test for categorial variables. Survival between groups was compared using the Log-Rank test and *p*-values < 0.05 were considered statistically significant. Additionally, Cox proportional hazards regression models were used to investigate the influence of different variables on one-year patient survival, death-censored graft survival, and overall graft survival. The univariate analysis included donor age (as a continuous variable as well as a categorial variable (≥65–69 vs. ≥70)), recipient age, HCV status, cold and warm ischemia time, labMELD, PNF, BPAR, re-operation, number of readmissions, and stay at ICU. Thereafter, a stepwise selection was used to identify the relevant variables in a multivariable Cox regression analysis. Results are shown as hazard ratios (HR) with a 95% confidence interval (CI) and *p*-value of the likelihood ratio test. All statistical analyses were performed with SAS 9.4, SAS Institute, Cary, NC, USA.

## 3. Results

### 3.1. Study Population Characteristics

Four hundred and six patients were included in the primary explorative analysis, representing all patients who received an oLT between January 2006 and May 2018 at the Department of General, Visceral and Transplant Surgery, University Hospital Muenster, Germany. The average donor age in the overall cohort was 51.9 ± 15.1 years, the youngest donor being 12 and the oldest, 84. Patients were then stratified based on donor age with a cut-off point of 65 years. Applying this strategy, it was found that 314 (77.3%) patients received grafts from a donor younger than 65, while 92 (22.7%) patients were transplanted with a graft from a donor ≥ 65 years old. When comparing baseline recipient characteristics (Table 1), significant differences were found for recipient age (<65: 51.5; ≥65: 55.4, *p* = 0.005) as well as the number of HU oLTs (<65: 22; ≥65: 1, *p* = 0.037). Next, the 92 patients who received a liver graft from donors ≥ 65 were further stratified based on the donor age into two groups: (1) ≥65–69 (52 patients, 12.8%) and (2) ≥70 (40 patients, 9.8%) years (Table 2). While the two groups were comparable regarding the baseline recipient characteristics (age, gender, and BMI), differences were found for underlying disease as an indication for oLT, although not reaching a level of significance: among the younger donor group, fewer recipients were suffering from HCC (17.3% vs. 30%; *p* = 0.073) and alcoholic cirrhosis (15.3% vs. 35%; *p* = 0.073), whereas more cases had ALF (13.5% vs. 2.5%; *p* = 0.073). In addition, the mean labMELD score, warm and cold ischemia times, as well as the percentage of HCV-positive donors showed no differences between the two groups (Table 2).

**Figure 1:**
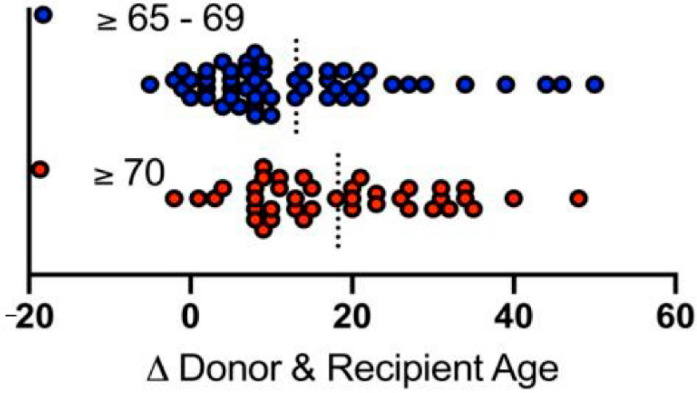
Age differences between donors and recipients. Blue dots represent donors of ≥65–69 years, red dots represent donors ≥ 70 years. Dashed bars indicate medians.

Donor age was 66.8 ± 1.5 years in donors ≥ 65–69 and 74.7 ± 3.7 in donors ≥ 70 (*p* < 0.001). Accordingly, younger donors had significantly lower DRI (Table 3). The average age difference between donor and recipient was 12.1 ± 10.7 years (donor ≥65–69, ranging from −5 to +41) and 18.4 ± 11.5 years (donor ≥ 70, ranging from −2 to +48, *p* = 0.044, Figure 1). During the study period, there was no age-specific protocol for pre-transplant graft biopsies.

### 3.2. One-Year Patient and Graft Survival

**Figure 2:**
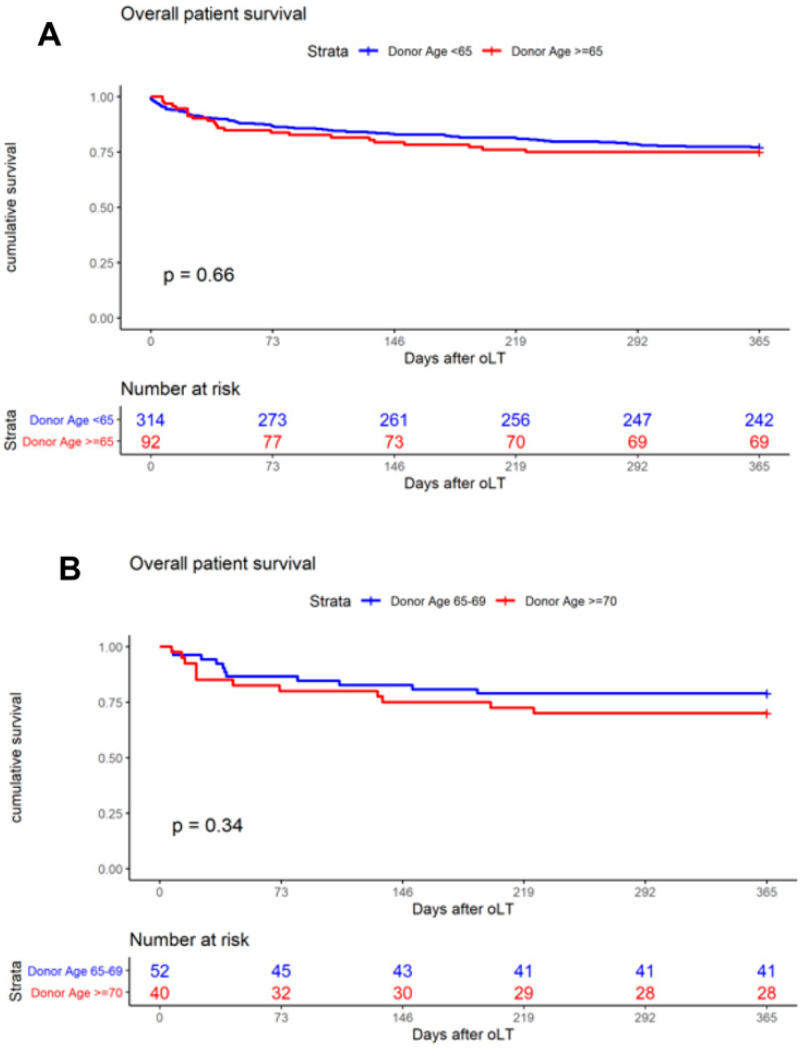
Kaplan–Meier curves demonstrate the impact on one-year patient survival of (**A**) donor age < 65 and ≥ 65 years; (**B**) donor age ≥ 65–69 and ≥ 70 years.

**Figure 3:**
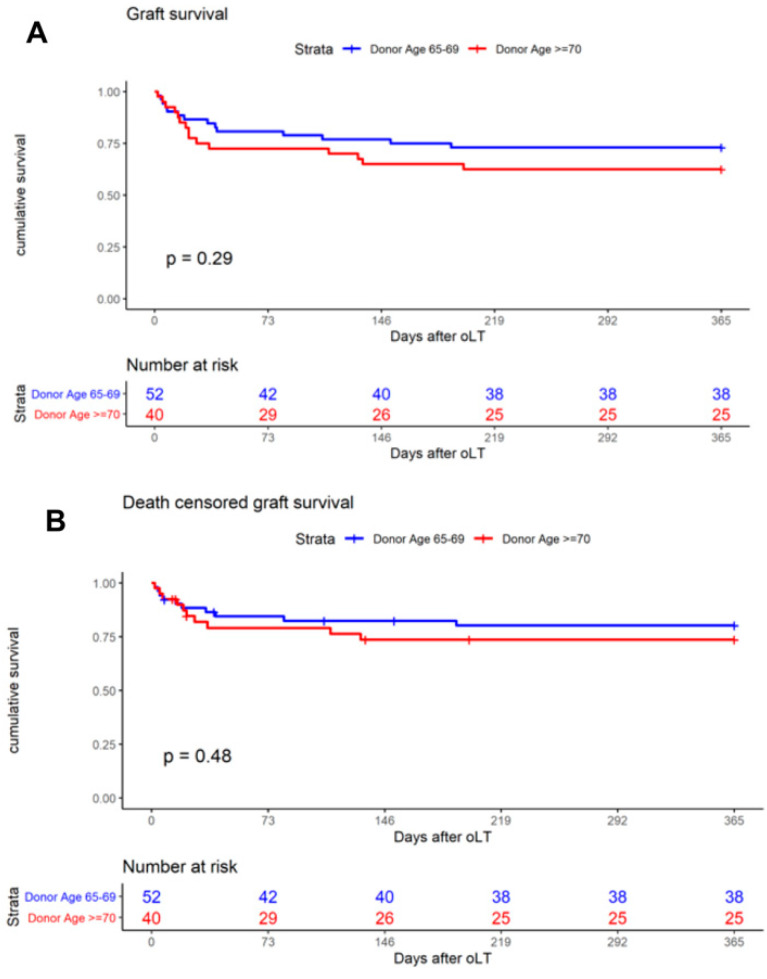
Kaplan–Meier curves demonstrate the impact on one-year graft survival of (**A**) donor age ≥65–69 and ≥ 70 years and (**B**) on one-year death-censored graft survival of donor age ≥65–69 and ≥ 70 years.

The primary outcome was one-year patient survival, and the Kaplan–Meier analysis was used to generate survival curves. When analyzing the entire cohort of 406 patients, one-year patient survival was found to be 77.1% (donor < 65) and 75.0% (donor ≥ 65, *p* = 0.66, Figure 2A), respectively. The analysis of age-stratified groups with donor ≥ 65 revealed one-year patient survival to be 78.9% (donor ≥ 65–69) and 70.0% (donor ≥ 70, *p* = 0.34, Figure 2B). A comparison of patient survival at day 30 (donor ≥65–69: 94.2%; donor ≥ 70: 85.0%) and day 90 (donor ≥ 65–69: 84.6%; donor ≥ 70: 80.0%) revealed no significant difference between the groups (Table 4). Overall, one-year graft survival was 73.1% (donor ≥ 65–69) and 62.5% (donor ≥ 70, *p* = 0.29, Figure 3A). Death-censored graft survival was 80.3% (donor ≥ 65–69) and 73.4% (donor ≥ 70, *p* = 0.48, Figure 3B). An analysis of the entire cohort of 406 patients indicated the overall one-year graft survival to be 72.93% (donor < 65) and 68.4% (donor ≥ 65, *p* = 0.370).

Next, unadjusted univariate Cox proportional hazard modeling was used to identify the influence of different variables on one-year patient survival (Table 5), overall graft survival (Table 6), and death-censored graft survival (Table 6). For one-year patient survival, neither categorial donor age (HR 1.054, 95% CI: 0.976–1.139, *p*-value = 0.1808) nor donor age ≥ 65–69 in comparison to donor age ≥ 70 (HR 0.671, 95% CI: 0.296–1.521, *p* = 0.3395) were found to be significantly associated with the endpoint. However, re-operation and the number of readmissions revealed a significant influence on one-year patient survival (Table 5). In addition, when analyzing one-year graft survival, donor age ≥ 65–69 was found to have a protective effect on graft loss at 365 days with an HR of 0.270 (95% CI: 0.102–0.711), when adjusted for potential confounders, revealing a negative influence of donor age ≥ 70 on graft survival (Table 6). Moreover, when one-year death-censored graft survival was analyzed, donor age ≥ 70 was again found to be significantly associated with the endpoint (HR for ≥ 65–69 vs. ≥ 70 0.215) (95% CI: 0.067–0.688) (Table 7).

### 3.3. Additional Outcome Parameters

PNF (donor ≥ 65–69: 15.3%; donor ≥ 70: 7.5%, *p* = 0.337), BPR (donor ≥ 65–69: 19.2%; donor ≥ 70: 15.0%, *p* = 0.782), and rates of re-transplantation (donor ≥ 65–69: 9.6%; donor ≥ 70: 20%, *p* = 0.227) within the first year were all comparable between the two groups (Table 4). Indications for re-oLT were PNF (donor ≥ 65–69: 100%, donor ≥ 70: 62.5%), as well as hepatic artery thrombosis (donor ≥ 70: 37.5%). Since re-LT patients are at a higher risk of death than first LT patients, we conducted a subgroup analysis excluding all re-LT patients and found one-year patient survival at 77.55% (donor ≥ 65–69) and 75.75% (donor ≥ 70), respectively. In addition, no differences were found when analyzing the frequency of EAD (donor ≥ 65–69: 41.17%; donor ≥ 70: 42.50% (*p* = 0.898), peak enzyme levels (AST and ALT), frequency of ITBL (donor < 65: 3.93%; donor ≥ 65, and 5%, *p* = 0.960), number of reoperations, and initial hospital stay or stay at ICU (Table 4).

### 3.4. Effect of CIT

**Figure 4:**
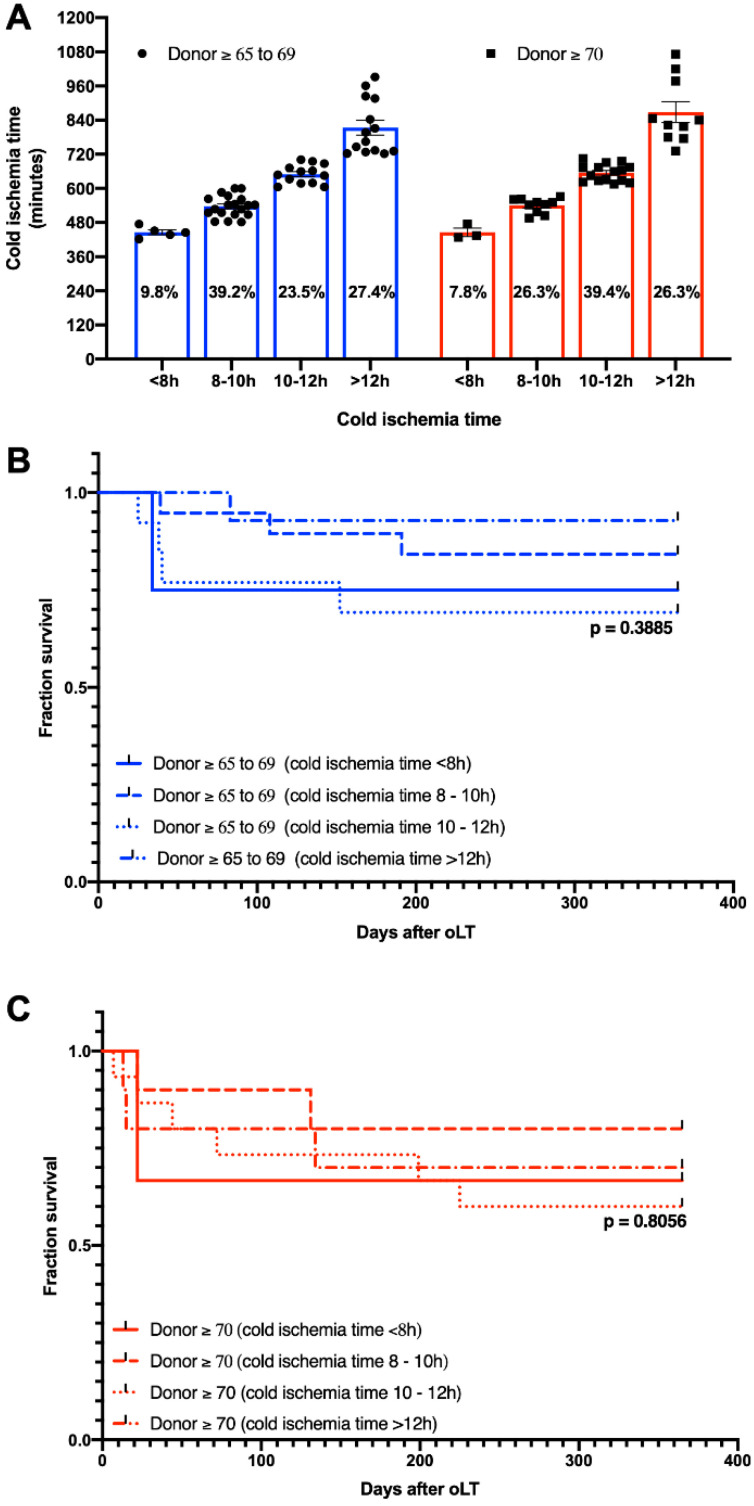
(**A**) Distribution of cold ischemia subcategories and Kaplan–Meier curves demonstrating the impact of cold ischemia time of one-year patient survival in (**B**) donor age ≥ 65–69 years and (**C**) donor age ≥ 70 years.

CIT was classified into four categories (<8 h, 8–10 h, 10–12 h, >12 h) according to Cassuto et al. [11] and the distribution among the age-stratified groups with donors ≥ 65 was analyzed. Most grafts from donors ≥ 65–69 were transplanted after a CIT of 8–10 h (39.2%), while the majority of grafts from donors ≥ 70 were transplanted after a CIT of 10–12 h (39.4%) (Figure 4A). Next, one-year patient survival was analyzed, and groups were stratified for CIT. No differences were found for CIT subcategories among donors ≥ 65–69 (Figure 4B) or donors ≥ 70 (Figure 4C). In addition, no significant differences were found when matching CIT was analyzed across donor subgroups (<8 h: donor ≥65–69: 75%; donor ≥ 70: 66.7%, 8–10 h: donor ≥ 65–69: 84.2%; donor ≥ 70: 80%, 10–12 h: donor ≥ 65–69: 69.2%; donor ≥ 70: 60%, >12 h: donor ≥65–69: 92.9%; donor ≥ 70: 70%)

## 4. Discussions

It is debatable whether center-specific conclusions can be drawn from large multicenter prospective studies like the CTS, or analyses from national registries like UNOS [2,5]. Therefore, this study aimed to clarify the extent to which general considerations of high donor age in oLT can be applied to an LT program with donors exceeding 65 years, representing 22.6% and 9.8% being older than 70 years, in a single institution. Our cohort included fewer donors exceeding 70 years (9.8% vs. 13%) and 65 years (22.6% vs. 25.2%), compared to recently published data from the Eurotransplant region and the CTS [2,3]. As expected, our rate of utilization of septuagenarian donors was notably higher than that reported for the UNOS region (9.8% vs. 4.3%) [5]. Recipients’ baseline characteristics were in general comparable between both elderly donor age groups. One difference was the rate of ALF as an indication for oLT (13.5% (≥65–69 years) vs. 2.5% (≥70 years); *p* = 0.069) among the two elderly donor age categories, even though the difference was insignificant, most likely due to the limited number of cases. This finding is as per the CTS and Eurotransplant data and could be attributed to the prioritization of ALF candidates in the allocation. The percentage of HCV-positive recipients of grafts from donors exceeding 69 years was higher compared to recipients of grafts from donors of ≥65–69 years (17.5% vs. 11.5%, *p* = 0.55), without reaching the level of significance. Since DAAs were available only by the end of the study period, this finding runs counter to the traditionally avoided combination of HCV and older donors. With the introduction of DAAs, HCV-positive recipients achieve outcomes comparable to HCV-negative patients after oLT [12]. Moreover, it was recently shown that even the use of viremic HCV-positive donors in HCV-negative recipients does not, on the other hand, impair the outcome in the post-DAA era [13]. 

The higher rate of HCC recipients in the older category in our study is also as per CTS and Eurotransplant data. In our opinion, allocating older grafts to HCC recipients is justified, as it was shown that especially older HCC recipients show the least impairment of graft survival by receiving elderly grafts [2]. Allocating elderly grafts to specific indication categories, e.g., HCC, is a matter of ongoing debate and further analyses from large studies and registries might be necessary to clarify this issue with a focus on the individual benefit of older grafts for the recipient [14,15]. Unfortunately, the outstanding work by Haugen et al., revealing a substantial survival benefit for waitlist candidates accepting an elderly graft, could not provide benefit information for specific indications such as HCC [7]. In our population, grafts from donors exceeding 70 years have been allocated more frequently to HCC and alcoholic cirrhosis recipients, whereas ALF recipients preferably received livers from younger donors. Even though the differences did not reach the level of significance, it reflects our intended recipient selection policy at the time. Careful selection of recipients of older grafts appears reasonable to avoid adding risks resulting in unfavorable outcomes. Through these measures, even the use of octogenarian donors has been reported to provide results comparable to the ones achieved with younger donors [16,17,18]. For donors exceeding 70 years, Bertuzzo et al. even reported comparable 5-year graft survival rates in unselected recipients, compared to younger donors [19].

As our study did not reveal any entirely new, previously unreported findings, the interpretation of the data obviously does not lead to changes in the clinical routine. Nevertheless, as mentioned above, the acceptance of grafts in our program is decided based on individual consideration of recipient- and donor-based risk factors. Therefore, we believe that it is essential to know the exact impact of increased donor age in our individual circumstances. This is especially relevant, as we are confronted with a severe donor shortage, forcing us to commonly evaluate marginal grafts, which is reflected by the rather high DRI above 2 in our cohort. Notably, the impact of the routine implementation of normothermic machine perfusion (NMP) on the outcome of older grafts, and likewise marginal grafts, will be the future focus of the transplant community [20]. There is currently one randomized controlled trial that demonstrates reduced ischemia-reperfusion injury (IRI) in older liver grafts (donors ≥ 70 years) undergoing NMP [21]. Whether a reduced IRI translates into superior clinical outcomes (e.g., reduced PNF) remains unknown currently. However, it is undoubted that viability assessment during NMP can provide significant insights into the graft quality and prediction of liver function and thus might help to expand the donor pool by assessing older liver grafts under near-physiological conditions.

Since older liver grafts are highly susceptible to cold-storage-elicited injury, it is recommended to keep the CIT as short as possible (preferably below 8 h [22]), to minimize deleterious effects on the post-transplant outcome. The CIT (donor ≥65–69: 10.5 h, donor ≥ 70: 10.8 h) reported here is rather long compared to previous studies [23,24] and must be considered when comparing the results obtained. However, our analysis also shows that longer ischemia times can be accepted, even for older grafts, when the remaining risk factors are well balanced. In addition, our analysis found no evidence of a higher CIT susceptibility among donors exceeding 70 years, compared to donors ≥ 65–69 years of age, which indicates that the functional reserve and capacity of response and recovery in older liver grafts have no strict linear pattern. 

Our study has not obtained insights that are entirely different from the previously published work in this matter. One-year patient survival in our study was unimpaired comparing donors below 65 and 65–69 years (77.1% vs. 78.5%) The survival rates for recipients of grafts exceeding 70 years were inferior to those receiving grafts in the donor age range of 65–69 years, even though the difference was insignificant due to the limited numbers in this single-center analysis. Having established elevated donor age as a risk factor for graft failure in our Cox proportional hazards regression models, it appears conceivable that well-conducted risk balancing might mitigate donor-associated risk factors, since age-stratified Log-Rank analysis revealed no significant differences in graft survival between donors ≥ 65–69 and ≥70 years of age. Given the aforementioned observation that the impact of increasing donor age is linear over the entire age range, one has to conclude that differences are very minute in comparing 65–69 years old donors to those exceeding 70. Accordingly, the meta-analysis of Dasari et al. has not identified 70 years as a donor age threshold predicting impaired graft or patient survival rates in LT [24]. Confirming the linearity of the influence of donor age on the outcome in oLT in their recent analysis of Eurotransplant data, Pratschke et al. also concluded that there is no threshold of donor age for the prediction of graft failure in oLT [25].

## 5. Conclusions

In summary, our results confirm previous findings that an absolute cut-off age threshold for donors does not exist. Elevated donor age should rather be considered as one of the several parameters in the decision-making process for allocation and individualized acceptance of grafts for oLT. Elderly donors, especially in the range of 65–69 years, are substantially helping to reduce the burden of donor organ shortage and waitlist mortality.

## Figures and Tables

**Table 1:** Recipient characteristics.

	<65 (*n* = 314)	≥65 (*n* = 92)	*p*-Value
**Age** (mean ± SD)	51.5 ± 11.9	55.4 ± 10.3	0.005 ^a^
**Gender** (% males)	64.3	65.2	0.902 ^b^
**BMI** (mean ± SD)	26.00 ± 5.4	26.86 ± 4.7	0.051 ^a^
**Indications for transplant** (%)			0.455 ^b^
ALF	14.3	8.7	
HCC	21.0	22.8	
Viral hepatitis	12.7	10.8	
PSC, PBC, SSC	12.1	13.0	
Alcoholic cirrhosis	13.6	23.9	
Cirrhosis other	7.3	7.6	
Other	19.4	13.0	
**HCV** (% antibody positive)	16.5	14.1	0.631 ^b^
**Cold ischemia time** (min, mean ± SD)	616.1 ± 167.9	641.1 ± 143.9	0.245 ^a^
**Warm ischemia time** (min, mean ± SD)	40.4 ± 9.6	40.2 ± 8.1	0.968 ^a^
**labMELD** (mean ± SD)	22.5 ± 11.9	21.8 ± 11.9	0.722 ^a^
**HU status** (*n*)	22	1	0.037 ^b^
**Previous transplants** (% ≥1)	12.7	8.7	0.360 ^b^

Results are presented as mean ± standard deviation (SD) or relative frequencies. BMI: body mass index, HCV: hepatitis C virus, ALF: acute liver failure, HCC: hepatocellular carcinoma, PSC: primary sclerosing cholangitis, PBC: primary biliary cholangitis, SSC: secondary sclerosing cholangitis, MELD: model of end-stage liver disease, HU: high urgency, ^a^ Wilcoxon rank-sum test, ^b^ Fisher’s exact test.

**Table 2:** Recipient characteristics.

	≥65–69 (*n* = 52)	≥70 (*n* = 40)	*p*-Value
**Age** (mean ± SD)	54.7 ± 10.6	56.3 ± 9.9	0.462 ^a^
**Gender** (% males)	71.1	57.5	0.191 ^b^
**BMI** (mean ± SD)	26.89 ± 4.6	26.82 ± 4.8	0.793 ^a^
**Indications for transplant** (%)			0.073 ^b^
ALF	13.5	2.5	
HCC	17.3	30.0	
Viral hepatitis	11.5	10.0	
PSC, PBC, SSC	13.4	12.5	
Alcoholic cirrhosis	15.3	35.0	
Cirrhosis other	11.5	2.5	
Other	13.5	7.5	
**HCV** (% antibody positive)	11.5	17.5	0.548 ^b^
**Cold ischemia time** (min, mean ± SD)	632.9 ± 137.1	651.5 ± 153.4	0.580 ^a^
**Warm ischemia time** (min, mean ± SD)	40.6 ± 8.6	39.8 ± 7.5	0.726 ^a^
**labMELD** (mean ± SD)	23.4 ± 12.9	20.1 ± 10.3	0.299 ^a^
**HU status** (*n*)	1	0	0.066 ^b^
**Previous transplants** (% ≥1)	7.69	10.0	0.724 ^b^

Results are presented as mean ± standard deviation (SD) or relative frequencies. BMI: body mass index, HCV: hepatitis C virus, ALF: acute liver failure, HCC: hepatocellular carcinoma, PSC: primary sclerosing cholangitis, PBC: primary biliary cholangitis, SSC: secondary sclerosing cholangitis, MELD: model of end-stage liver disease, HU: high urgency, ^a^ Wilcoxon rank-sum test, ^b^ Fisher’s exact test.

**Table 3:** Donor characteristics.

	≥65–69 (*n* = 52)	≥70 (*n* = 40)	*p*-Value
**Age** (mean ± SD)	66.8 ± 1.5	74.7 ± 3.7	<0.0001 ^a^
**Gender** (% males)	63.5	50.0	0.209 ^b^
**BMI** (mean ± SD)	27.4 ± 3.9	26.6 ± 2.8	0.381 ^a^
**Donor Center** (% national)	86.5	87.5	1.000 ^b^
**DRI** (mean ± SD)	2.0 ± 0.2	2.2 ± 0.3	<0.0001 ^a^

Results are presented as mean ± standard deviation (SD) or relative frequencies. BMI: body mass index, DRI: donor risk index, ^a^ Wilcoxon rank-sum test, ^b^ Fisher’s exact test.

**Table 4:** Clinical outcome.

	≥65–69 (*n* = 52)	≥70 (*n* = 40)	*p*-Value
**Patient survival (%)**			0.335 ^b^
30 d	94.2	85.0	
90 d	84.6	80.0	
365 d	78.8	70.0	
**Graft survival (%)**			0.291 ^b^
30 d	86.5	75.0	
90 d	78.8	72.5	
365 d	73.1	62.6	
**Re-oLT within 1y** (%)	9.6	20.0	0.227 ^b^
**PNF** (%)	15.3	7.5	0.337 ^b^
**Biopsy-proven rejection** (%)	19.2	15.0	0.782 ^b^
**Re-operation** (%)	55.8	65.0	0.398 ^b^
**Number of reoperations** (median MIN, MAX)	1 (0.12)	1 (0.8)	0.101 ^a^
**EAD (%)**	41.17	42.50	0.898 ^b^
**Peak AST** (U/l)	5932.4 ± 7269.0	4576.5 ± 5045.4	0.878 ^a^
**Peak ALT** (U/l)	3453.5 ± 4528.9	2646.6 ± 3246.9	0.829 ^a^
**ITBL (%)**	3.93	5.00	0.960 ^b^
**Stay at ICU** (d, mean ± SD)	12.9 ± 17.7	13.8 ± 19.4	0.971 ^a^
**Initial hospital stay** (d, mean ± SD)	48.5 ± 33.0	47.2 ± 36.0	0.545 ^a^
**Number of readmissions** (mean ± median (MIN, MAX))	2 (0–10)	1 (0–8)	0.678 ^a^
**Length of readmissions** (d, median (MIN, MAX))	22.0 (0.122)	21 (0.191)	0.762 ^a^

Results are presented as mean ± standard deviation (SD), median (with minimal and maximal values), or relative frequencies. oLT: orthotopic liver transplant, PNF: primary non-function, AST: aspartate aminotransferase, EAD: early allograft dysfunction, ALT: alanine aminotransferase, ITBL: ischemic-type biliary lesions, ICU: intensive care unit, ^a^ Wilcoxon rank-sum test, ^b^ Fisher’s exact test.

**Table 5:** Cox proportional hazards regression model with univariate and multivariable Cox regression analyses of one-year patient survival for recipients of grafts ≥ 65 years.

	Univariate		Multivariate	
	HR (95% CI)	*p*-Value	HR (95% CI)	*p*-Value
**Donor age** (continuous variable)	1.054 (0.976–1.139)	0.1808		
**Donor age** (≥65–69 vs. ≥70)	0.671 (0.296–1.521)	0.3395		
**Recipient age**	0.992 (0.954–1.032)	0.7017		
**HCV** (positive vs. negative)	1.825 (0.428–7.783)	0.4163		
**Cold ischemia time**	1.000 (0.997–1.003)	0.9076		
**Warm ischemia time**	1.039 (0.988–1.092)	0.1325		
**labMELD**	1.011 (0.975–1.048)	0.5520		
**PNF** (yes vs. no)	5.660 (2.307–13.883)	0.0002		
**Biopsy-proven rejection** (yes vs. no)	1.874 (0.738–4.756)	0.1865		
**Re-operation** (yes vs. no)	18.694 (2.518–138.808)	0.0042	23.971 (3.163–181.638)	0.0021
**Number of readmissions**	0.331 (0.188–0.584)	0.0001	0.308 (0.171–0.554)	<0.0001
**Stay at ICU**	1.029 (1.016–1.041)	<0.0001		

HR: hazard ratios, CI: 95% confidence interval. HCV: hepatitis c virus, PNF: primary non-function, ICU: intensive care unit, MELD: model of end-stage liver disease.

**Table 6:** Cox proportional hazards regression model with univariate and multivariable Cox regression analyses of one-year graft survival for recipients of grafts ≥ 65 years.

	Univariate		Multivariate	
	HR (95% CI)	*p*-Value	HR (95% CI)	*p*-Value
**Donor age** (continuous variable)	1.032 (0.946–1.125)	0.4840		
**Donor age** (≥65–69 vs. ≥70)	0.732 (0.305–1.759)	0.4856	0.215 (0.067–0.688)	0.0096
**Recipient age**	1.019 (0.971–1.069)	0.4511		
**HCV** (positive vs. negative)	0.899 (0.263–3.067)	0.8644		
**Cold ischemia time**	1.001 (0.998–1.004)	0.6721		
**Warm ischemia time**	1.009 (0.956–1.066)	0.7400		
**labMELD**	1.017 (0.979–1.056)	0.3899		
**PNF** (yes vs. no)	49.862 (16.468–150.975)	<0.0001	70.749 (18.744–267.043)	<0.0001
**Biopsy-proven rejection** (yes vs. no)	0.863 (0.253–2.948)	0.8143		
**Re-operation** (yes vs. no)		0.9888		
**Number of readmissions**	0.668 (0.487–0.918)	0.0129		
**Stay at ICU**	1.029 (1.016–1.042)	<0.0001	1.036 (1.017–1.055)	0.0002

HR: hazard ratios, CI: 95% confidence interval. HCV: hepatitis c virus, PNF: primary non-function, ICU: intensive care unit, MELD: model of end-stage liver disease.

**Table 7:** Cox proportional hazards regression model with univariate and multivariable Cox regression analyses of one-year death-censored graft survival.

	Univariate		Multivariate	
	HR (95% CI)	*p*-Value	HR (95% CI)	*p*-Value
**Donor age** (continuous variable)	1.048 (0.977–1.125)	0.1887		
**Donor age** (≥65–69 vs. ≥70)	0.678 (0.327–1.404)	0.2952	0.270 (0.102–0.711)	0.0081
**Recipient age**	0.986 (0.952–1.021)	0.4351		
**HCV** (positive vs. negative)	1.384 (0.419–4.573)	0.5923		
**Cold ischemia time**	1.001 (0.998–1.003)	0.6600		
**Warm ischemia time**	1.019 (0.975–1.066)	0.4018		
**labMELD**	1.013 (0.981–1.045)	0.4266		
**PNF** (yes vs. no)	32.894 (12.940–83.620)	<0.0001	33.421 (10.391–107.490)	<0.0001
**Biopsy-proven rejection** (yes vs. no)	1.578 (0.674–3.699)	0.2935		
**Re-operation** (yes vs. no)	26.129 (3.550–192.310)	0.0014	10.182 (1.293–80.172)	
**Number of readmissions**	0.612 (0.458–0.817)	0.0009		
**Stay at ICU**	1.030 (1.019–1.041)	<0.0001	1.027 (1.010–1.043)	0.0015

HR: hazard ratios, CI: 95% confidence interval. HCV: hepatitis c virus, PNF: primary non-function, ICU: intensive care unit, MELD: model of end-stage liver disease.

## Data Availability

The datasets generated during and/or analyzed during the current study are available from the corresponding author on reasonable request.

## References

[1] Bodzin A.S., Baker T.B. (2018). Liver Transplantation Today: Where We Are Now and Where We Are Going. Liver Transplant..

[2] Houben P., Döhler B., Weiß K.H., Mieth M., Mehrabi A., Süsal C. (2020). Differential Influence of Donor Age Depending on the Indication for Liver Transplantation—A Collaborative Transplant Study Report. Transplantation.

[3] de Boer J.D., Blok J.J., Putter H., Koopman J.J.E., van Hoek B., Samuel U., van Rosmalen M., Metselaar H.J., Alwayn I.P.J., Guba M. (2018). Optimizing the Use of Geriatric Livers for Transplantation in the Eurotransplant Region. Liver Transplant..

[4] Feng S., Goodrich N., Bragg-Gresham J., Dykstra D., Punch J., DebRoy M., Greenstein S., Merion R. (2006). Characteristics Associated with Liver Graft Failure: The Concept of a Donor Risk Index. Am. J. Transplant..

[5] Halazun K., Rana A.A., Fortune B., Quillin R.C., Verna E.C., Samstein B., Guarrera J.V., Kato T., Griesemer A.D., Fox A. (2017). No country for old livers? Examining and optimizing the utilization of elderly liver grafts. Am. J. Transplant..

[6] Machicao V.I., Bonatti H., Krishna M., Aqel B.A., Lukens F.J., Nguyen J.H., Rosser B.G., Satyanarayana R., Grewal H.P., Hewitt W.R. (2004). Donor age affects fibrosis progression and graft survival after liver transplantation for hepatitis C1. Transplantation.

[7] Haugen C.E., Bowring M.G., Holscher C.M., Jackson K.R., Garonzik-Wang J., Cameron A.M., Philosophe B., McAdams-DeMarco M., Segev D.L. (2019). Survival benefit of accepting livers from deceased donors over 70 years old. Am. J. Transplant..

[8] Olthoff K.M., Kulik L., Samstein B., Kaminski M., Abecassis M., Emond J., Shaked A., Christie J.D. (2010). Validation of a current definition of early allograft dysfunction in liver transplant recipients and analysis of risk factors. Liver Transplant..

[9] Becker F., Vogel T., Voß T., Mehdorn A.-S., Schütte-Nütgen K., Reuter S., Mohr A., Kabar I., Bormann E., Vowinkel T. (2018). The weekend effect in liver transplantation. PLoS ONE.

[10] Becker F., Voß T., Mohr A., Mehdorn A.-S., Schütte-Nütgen K., Reuter S., Kabar I., Bormann E., Vowinkel T., Palmes D. (2019). Impact of nighttime procedures on outcomes after liver transplantation. PLoS ONE.

[11] Cassuto J.R., Patel S.A., Tsoulfas G., Orloff M.S., Abt P.L. (2008). The Cumulative Effects of Cold Ischemic Time and Older Donor Age on Liver Graft Survival. J. Surg. Res..

[12] Cotter T.G., Paul S., Sandıkçı B., Couri T., Bodzin A.S., Little E.C., Sundaram V., Charlton M. (2019). Improved Graft Survival After Liver Transplantation for Recipients With Hepatitis C Virus in the Direct-Acting Antiviral Era. Liver Transplant..

[13] Cotter T.G., Paul S., Sandıkçı B., Couri T., Bodzin A.S., Little E.C., Sundaram V., Charlton M. (2019). Increasing Utilization and Excellent Initial Outcomes Following Liver Transplant of Hepatitis C Virus (HCV)-Viremic Donors Into HCV-Negative Recipients: Outcomes Following Liver Transplant of HCV-Viremic Donors. Hepatology.

[14] Durand F., Francoz C. (2020). Optimizing Allocation of Older Donors in Liver Transplantation: The Objectives of Allocation Policies Also Matter. Transplantation.

[15] Moosburner S., Ritschl P.V., Wiering L., Gassner J.M.G.V., Öllinger R., Pratschke J., Sauer I.M., Raschzok N. (2019). High donor age for liver transplantation: Tackling organ scarcity in Germany. Der Chirurg Zeitschrift fur Alle Gebiete der Oper. Med..

[16] Gastaca M., Guerra M., Martinez L.A., Ruiz P., Ventoso A., Palomares I., Prieto M., Matarranz A., Valdivieso A., de Urbina J.O. (2016). Octogenarian Donors in Liver Transplantation. Transplant. Proc..

[17] Biancofiore G., Bindi M., Ghinolfi D., Lai Q., Bisa M., Esposito M., Meacci L., Mozzo R., Spelta A., Filipponi F. (2017). Octogenarian donors in liver transplantation grant an equivalent perioperative course to ideal young donors. Dig. Liver Dis..

[18] Martín L.G., Grande A.M., Roux D.P., Cibrián C.G., Martín C.F., Gandía M.R., Buenadicha A.L. (2018). Short-term Results of Liver Transplantation With Octogenarian Donors. Transplant. Proc..

[19] Bertuzzo V.R., Cescon M., Odaldi F., Di Laudo M., Cucchetti A., Ravaioli M., Del Gaudio M., Ercolani G., D’Errico A., Pinna A.D. (2017). Actual Risk of Using Very Aged Donors for Unselected Liver Transplant Candidates: A European Single-center Experience in the MELD Era. Ann. Surg..

[20] Becker F., Pascher A., Brockmann J.G. (2020). Maschinenperfusion zur Konditionierung der Leber und Niere vor Transplantation. Der Chirurg Zeitschrift fur Alle Gebiete der Oper. Med..

[21] Ghinolfi D., Rreka E., De Tata V., Franzini M., Pezzati D., Fierabracci V., Masini M., Insilla A.C., Bindi M.L., Marselli L. (2019). Pilot, Open, Randomized, Prospective Trial for Normothermic Machine Perfusion Evaluation in Liver Transplantation From Older Donors. Liver Transplant..

[22] Segev D.L., Maley W.R., Simpkins C.E., Locke J.E., Nguyen G.C., Montgomery R.A., Thuluvath P.J. (2007). Minimizing risk associated with elderly liver donors by matching to preferred recipients. Hepatology.

[23] Haugen C.E., Thomas A., Garonzik-Wang J., Massie A.B., Segev D.L. (2018). Minimizing Risk Associated With Older Liver Donors by Matching to Preferred Recipients: A National Registry and Validation Study. Transplantation.

[24] Dasari B.V., Mergental H., Isaac J.R., Muiesan P., Mirza D.F., Perera T. (2017). Systematic review and meta-analysis of liver transplantation using grafts from deceased donors aged over 70 years. Clin. Transplant..

[25] Pratschke S., Bender A., Boesch F., Andrassy J., van Rosmalen M., Samuel U., Rogiers X., Meiser B., Küchenhoff H., Driesslein D. (2018). Association between donor age and risk of graft failure after liver transplantation: An analysis of the Eurotransplant database. Transpl. Int..

